# Liver PET Reloaded: Automated Synthesis of [^68^Ga]Ga-BP-IDA for Positron Imaging of the Hepatobiliary Function and First Clinical Experience

**DOI:** 10.3390/diagnostics13061144

**Published:** 2023-03-16

**Authors:** Anke Werner, Martin Freesmeyer, Christian Kühnel, Robert Drescher, Julia Greiser

**Affiliations:** Clinic of Nuclear Medicine, Jena University Hospital, Am Klinikum 1, 07747 Jena, Germany

**Keywords:** PET/CT, hepatobiliary imaging, liver function, IDA derivatives, dynamic PET, HCC

## Abstract

Hepatobiliary scintigraphy is a well-established nuclear imaging method for evaluating liver function and displaying the biliary system, but the spatial and temporal resolution is limited, and, there is still no established PET equivalent. Adapted from the work of Schuhmacher et al. in 1983, the production of a ^68^Gallium-labeled substitute, tetrabromophthalein ([^68^Ga]Ga-BP-IDA), was undertaken according to current Good Manufacturing Practice (GMP) standards and proved feasible and reproducible. PET/CT with the radiotracer was performed in two complex patients with hepatocellular carcinoma in preparation for transarterial radioembolization. Due to its high spatial and temporal resolution, localization of areas with impaired liver function and visualization of the biliary system were possible. We could demonstrate that this ^68^Gallium-labeled, IDA-based PET tracer is feasible and could advance hepatic and biliary function PET imaging.

## 1. Introduction

A general trend in nuclear medicine is the translation of classical scintigraphic methods to positron emission tomography/computed tomography (PET/CT)-based image acquisition, due to its superior temporal and spatial resolution, and better quantification ability. Examples are the addition of ^18^F sodium fluoride PET/CT to bone scintigraphy, of ^124^I PET/CT to ^131^I thyroid scintigraphy, and the replacement of ^67^Ga citrate by ^68^Ga-based PET tracers. Still, there is no established PET substitute for hepatobiliary scintigraphy, which would realize its advantages.

Hepatobiliary scintigraphy is a non-invasive nuclear imaging method for the evaluation of hepatobiliary function and the excretory biliary system. For many years, ^99m^Tc-marked iminodiacetic acid (IDA) agents were used in cholescintigraphy for the diagnosis of various biliary diseases and proved excellent for early diagnosis prior to anatomical changes in the hepatobiliary system, since distinguishable anatomic alterations may appear temporally delayed in comparison to physiologic changes [1]. Furthermore, the data acquisition provided quantitation of both regional and global liver diseases. Over time, several modifications to the original IDA agents were introduced. [^99m^Tc]Tc-mebrofenin (also referred to as BrIDA) as well as [^99m^Tc]Tc-lidofenin (HIDA) are considered very suitable agents for hepatic and biliary diagnostic methods due to their pharmacokinetic attributes, particularly in the diagnosis of acute and chronic gallbladder diseases, biliary leaks and obstruction, as well as biliary atresia [2]. Ekman et al. first described the quantitative determination of the hepatic uptake function by calculating the clearance rate of another IDA analog, IODIDA, from the blood [3]. In 2010, de Graaf et al. reported about hepatic ^99m^Tc-mebrofenin uptake rate measurements using dynamic scintigraphy and calculating liver uptake of mebrofenin as an increase in blood-pool-corrected ^99m^Tc-mebrofenin uptake per minute over a period of 200 s. In their study, they appreciated the method as a useful test for the assessment of liver function in liver surgery and liver transplantation [2]. Nevertheless, as mentioned above, scattered radiation in ^99m^Tc-based single-photon emission diagnostics may be a crucial disadvantage in terms of the accuracy of measurements and image quality. The spatial and temporal resolutions of scintigraphic methods are limited. Images with high temporal resolution are typically planar, but the two-dimensional images do not have the ability to evaluate detailed liver function on a segmental level [2]. The lack of anatomical mapping can be overcome with single photon emission computed tomography (SPECT)/CT technology, although it is still slow and does not allow exact activity quantification.

As early as 1983, Schuhmacher et al. described the chemical synthesis of an IDA-based radiopharmaceutical for the quantitative study of hepatobiliary function, ^68^Ga-labeled tetrabromophthalein ([^68^Ga]Ga-BP-IDA) [4]. In their study, they described the feasibility of the synthesis of the tracer, its evaluation in animal experiments, and its successful use in two healthy volunteers. However, the idea was not further developed, probably because of the limited availability of PET technology at the time, the elaborate tracer synthesis, as well as the fast advancement of CT and magnetic resonance (MR) methods, which were then used in clinical practice.

In this project, we established a production protocol for [^68^Ga]Ga-BP-IDA in accordance with Good Manufacturing Practice (GMP) standards in a certified laboratory, since PET/CT scanners are now widely used in many countries and the technology is fast and reliable. PET/CT with [^68^Ga]Ga-BP-IDA was performed in two particularly difficult individual cases in the preparatory process for transarterial radioembolization (TARE).

## 2. Materials and Methods

### 2.1. Radiolabeling Procedure of [^68^Ga]Ga-BP-IDA and Quality Control

**Figure 1:**
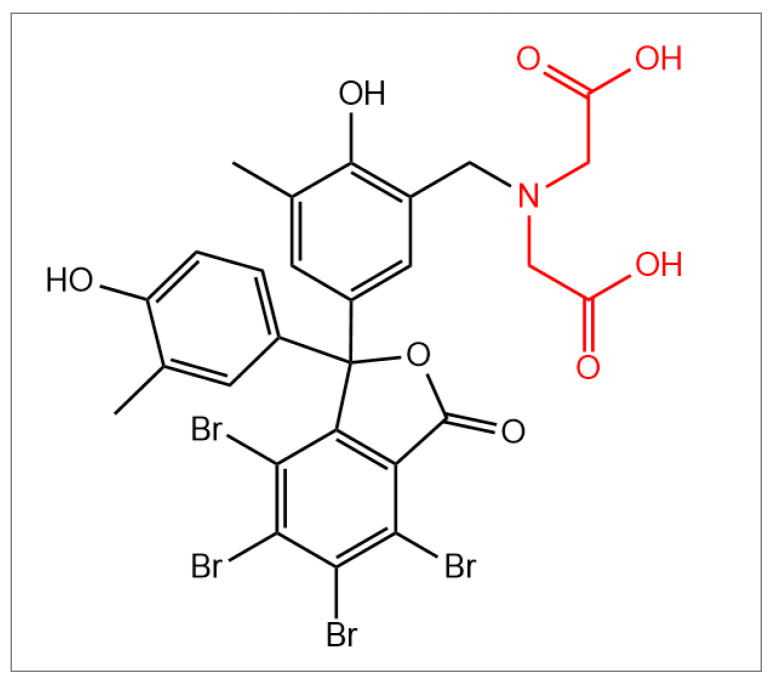
Structural formula of BP-IDA, with methyliminodiacetic acid (IDA) colored in red and the substituted tetrabromophthalein in black.

All radiopharmaceutical syntheses and quality control steps were carried out in compliance with GMP standards. 4,5,6,7-Tetrabromo-*o*-cresolphthalein-3′-methyliminodiacetic acid (BP-IDA, Figure 1) was provided by Sirius Fine Chemicals GmbH (Bremen, Germany).

Immediately before the synthesis, an aliquot of BP-IDA was dissolved in a 0.1 M sodium hydroxide solution, yielding a purple stock solution with a concentration of 150 µg/mL. For the synthesis, 30 µg of BP-IDA (in 200 µL of stock solution) was added to 3 mL of a 1.5 M HEPES (4-(2-hydroxyethyl)-1-piperazineethanesulfonic acid) buffer solution (pH 4.5). [^68^Ga]Ga-chloride radiolabeling solution was eluted with 0.1 M hydrochloric acid from a radionuclide generator (GalliaPharm^®^; Eckert & Ziegler, Berlin, Germany). The automated synthesis of [^68^Ga]Ga-BP-IDA was performed on a Scintomics GRP synthesizer (SCINTOMICS Molecular, Applied Theranostics Technologies GmbH, Fürstenfeldbruck, Germany) using sterile reagents and disposable cassettes for labeling ^68^Ga-peptides (ABX advanced biochemical compounds, Radeberg, Germany). Following the elution and purification of the ^68^Ga labeling solution via a PS-H^+^ cartridge, the eluate and the HEPES buffer containing BP-IDA were combined and heated to 100 °C for 10 min. Subsequently, solid-phase extraction (SPE) cartridge purification of the crude product was performed using a C8 Light SPE cartridge (Macherey Nagel, Düren, Germany) and 2 mL of an aqueous ethanol solution (60% *v*/*v*) for elution. The final product underwent sterile filtration and was diluted with 16 mL of unbuffered 0.9% saline. [^68^Ga]Ga-BP-IDA is not stable in phosphate buffered saline (PBS) solution as it exhibits rapid demetallation. In saline solution (0.9%), the tracer is stable for at least two hours after production.

Quality control of the product was carried out according to the standard requirements for ^68^Ga tracers in the European Pharmacopeia (Ph. Eur.). The radiochemical purity (RCP) was determined via radio high pressure liquid chromatography (HPLC) using a C18 reversed-phase column (125 mm × 4 mm) and the following gradient: flow of 1 mL/min, 0.0–2.5 min 97.0% solvent A (water/trifluoroacetic acid; 99.9%/0.1%) and 3.0% solvent B (acetonitrile/trifluoroacetic acid; 99.9%/0.1%), 2.5–10.0 min 97.0% → 0.0% solvent A, 10.0–13.0 min 0.0% solvent A, and 100% solvent B. The signal of [^68^Ga]Ga-BP-IDA appeared at 8.0 min and was always ≥95.0%. Additionally, the RCP was determined via radio thin layer chromatography (TLC), using a silica gel plate strip (70 mm × 10 mm) and a 0.1 M sodium acetate solution. The activity at R_f_ = 0.0 was assigned to [^68^Ga]Ga-BP-IDA, and the activity at R_f_ = 1.0 to non-bound, aqueous ^68^Ga(III). The determination of ^68^Ga in colloidal form as specified in the Ph. Eur. (using a glass fiber plate and ammonium acetate/methanol solution) was not possible, as the signal of [^68^Ga]Ga-BP-IDA remained at R_f_ = 0.0 along with the colloid.

### 2.2. PET/CT Protocol

In patients scheduled for TARE (40–50 cases per year in our institution), preparatory examinations are performed to anticipate the decrease in liver function that could be caused by radioembolization, thus minimizing the risk of liver failure. Serological markers and the Liver Maximum Capacity Test (LiMAx) reflect global liver function only. Therefore, in two individually selected, clinically complex patients with hepatocellular carcinoma (HCC), [^68^Ga]Ga-BP-IDA PET/CT was performed to facilitate a more detailed preinterventional assessment of regional liver function to estimate the risks of different TARE approaches regarding liver function deterioration, i.e., to decide if a higher microsphere activity can be injected that would lead to a higher tumor dose, but also to a higher level of liver damage. The clinical indications for PET/CT were confirmed in a multidisciplinary setting. Patients gave informed consent. The recommended diagnostic reference value for ^68^Ga-PET tracers in Germany is 2.0–2.5 MBq/kg body weight, which was adhered to for [^68^Ga]Ga-BP-IDA to ensure the sufficient image quality of the PET acquisition.

The patients were placed in a supine position in a Biograph mCT 40 PET scanner (Siemens Healthineers, Erlangen, Germany). A scout image of the upper abdomen for the required bed position was acquired. For attenuation correction, an unenhanced low-dose CT (50 mAs; 120 kV) of the upper abdomen was performed. CT images were reconstructed with a 3 mm slice thickness. Early dynamic PET was carried out in list mode, with acquisition starting simultaneously with the i.v. administration of the radiotracer bolus and continuing for 10 and 20 min in patients A and B, respectively. Only in patient A was an unenhanced whole-body low-dose CT (50 mAs; 120 kV) for attenuation correction followed by static whole-body acquisitions starting 18, 30, 45, and 60 min after radiotracer administration (acquisition time: 45 s/bed position) performed.

Image reconstruction was performed with the TrueX HD software (Siemens Healthineers, Erlangen, Germany) with 3D attenuation-weighted ordered subsets and expectation maximization at four iterations, 12 subsets with a 5-mm post-reconstruction gaussian filter, attenuation image segmentation, and a 512 × 512 pixel matrix. The acquired list mode (dynamic PET) data were reconstructed in subsequent 60-s time intervals (frames).

### 2.3. Image Analysis

Organ distribution of the radiotracer was determined in patient A by manually contouring the liver, gall bladder, kidneys, spleen, skeletal system, intestines, urinary bladder, and peripheral vein cannula (containing residual activity after tracer injection) in the CT for the attenuation correction using the PMOD software package (PMOD v4.0, PMOD Technologies LLC, Zurich, Switzerland). After subtracting remnant radiotracer activity in the peripheral venous catheter from the total prepared activity, the relative organ distribution (in %) could be calculated in relation to the determined organ volume in milliliter at 14 different time points (0.5, 1.5, 2.5, 3.5, 4.5, 5.5, 6.5, 7.5, 8.5, 9.5, 18, 30, 45, and 60 min after radiotracer injection). Mean and maximal standardized uptake values (SUV_mean_ and SUV_max_) were determined by manually placing volumes of interest (VOIs) over representative areas in the liver and gall bladder at the above-mentioned 14 time points and in the kidneys, spleen, skeletal system, intestines, and urinary bladder at 18, 30, 45, and 60 min after radiotracer injection. Additionally, VOIs were placed in the HCC tumor lesions and non-tumorous liver tissue to determine SUV_max_ and SUV_mean_. Measurements were performed on a syngo.via workstation (MM Oncology module, Siemens Healthineers, Erlangen, Germany).

## 3. Results

### 3.1. Tracer Preparation and Quality Control Results

The radiotracer preparation was successful in all cases. All quality control results were within the specified limits (Table 1). The pH of the final solution was 6.5 ± 0.7, the ethanol content was 6.1% ± 0.4% (*v*/*v*). The RCP was determined to be ≥95.0% using radio TLC and radio HPLC, although the Ph. Eur. method for the detection of colloidal ^68^Ga cannot be applied due to the significant retention of [^68^Ga]Ga-BP-IDA at the bottom of the TLC plate. However, the SPE purification step during the radiosynthesis should reduce any ^68^Ga colloid content to a minimum. Using the TLC method according to Ph. Eur., the HEPES content of the final solution was verified to be below 200 µg per total volume. The endotoxin content was ≤0.5 EU/mL. All samples were successfully tested for sterility.

### 3.2. Tracer Dynamics and Distribution on PET/CT

**Figure 2:**
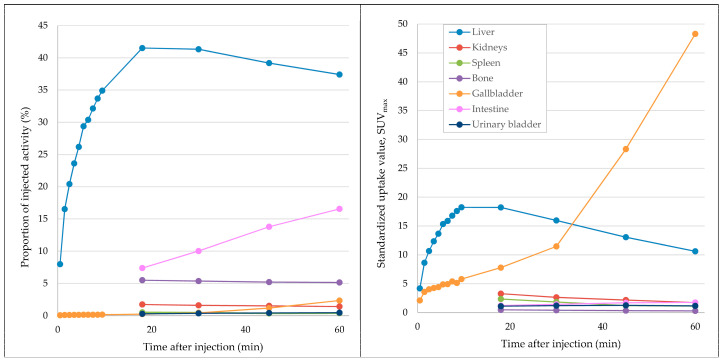
Distribution (left diagram) and uptake values (right diagram) of ^68^Ga-BP-IDA in organs on early-dynamic (0–10 min) and static PET/CT images (18/30/45/60 min) of patient A. ^68^Ga-BP-IDA is primarily accumulated in the liver and excreted with the bile. (The increase in SUV_max_ in the gall bladder almost immediately after tracer injection is caused by scattered radiation from adjacent liver tissue due to the high positron energy of ^68^Ga).

**Figure 3:**
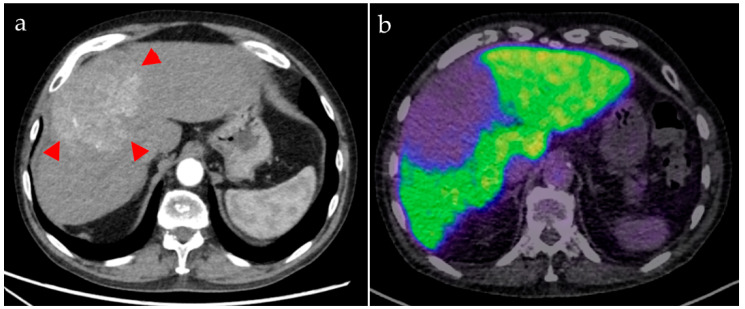
Patient A. The contrast-enhanced CT (a, arterial phase) shows the hypervascularized HCC ((**a**), arrowheads), involving both liver lobes. [^68^Ga]Ga-BP-IDA PET/CT (**b**), acquired 10 min after tracer injection, shows low uptake in the tumor (SUV_max_ 4.9) compared with healthy liver tissue (SUV_max_ 18.2).

**Figure 4:**
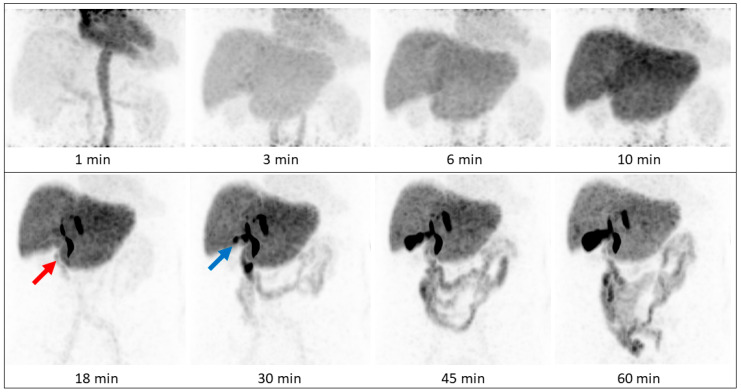
Patient A, maximum intensity projections (MIPs) of the upper abdomen. [^68^Ga]Ga-BP-IDA distribution on early dynamic (upper row) and static acquisitions (lower row, whole body acquisitions). Tracer passes into the duodenum (red arrow) before appearing in the gallbladder (blue arrow). Times are after i.v. tracer injection.

**Figure 5:**
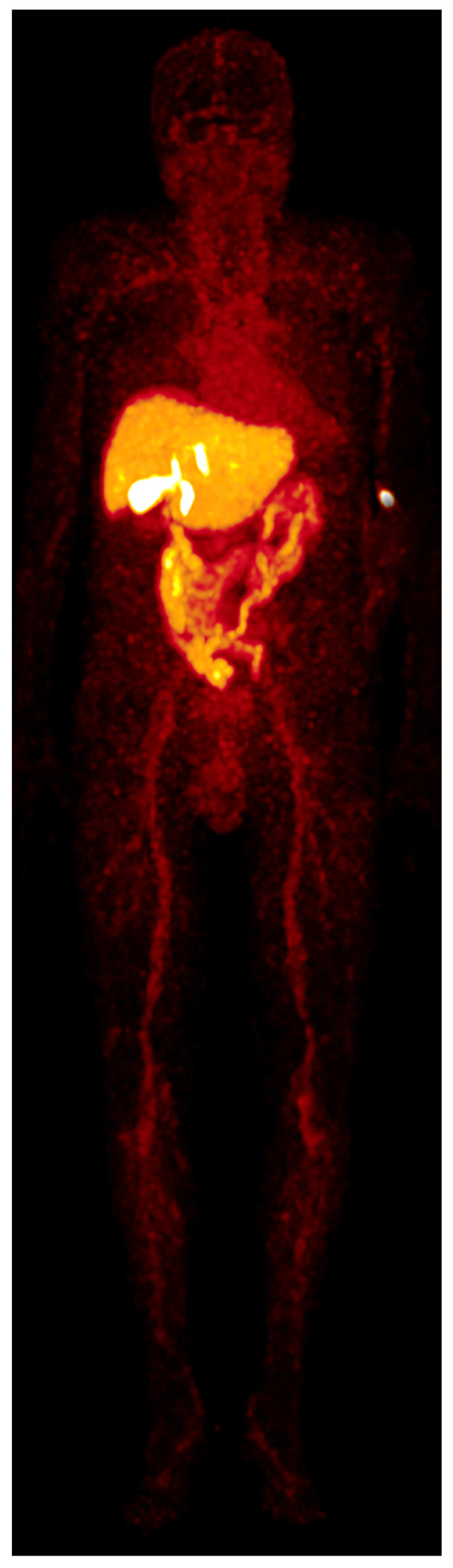
Whole-body MIP was acquired 60 min after the radiotracer injection, patient A. No relevant tracer uptake is seen in the bones, brain, or kidneys. Injection site at the left arm.

Two patients (both male; aged 72 and 82 years) underwent [^68^Ga]Ga-BP-IDA-PET/CT examinations. Activities of 144 MBq and 166 MBq [^68^Ga]Ga-BP-IDA, respectively, were prepared and injected intravenously. No immediate or delayed complications occurred. After intravenous administration, [^68^Ga]Ga-BP-IDA showed rapid liver tissue accumulation with a maximum of 41.5% of the injected activity (SUV_max_ 18.2) (Figure 2, Figure 3 and Figure 4). Biliary excretion was evident after 15 min with passage to the common hepatic duct. Tracer accumulation in the gall bladder was initially seen after 18 min, but continued to increase further, up to a SUV_max_ of 48.3 after 60 min. After 30 min, the transition to the duodenum and jejunum had already begun (Figure 4). Liver tissue uptake started to decrease slowly after 18 min, but still represented 37.4% of the administered activity after 60 min. No relevant uptake was seen in the spleen or the skeletal system. The radiotracer showed no significant urinary excretion (maximum kidney uptake of 1.7% of the administered activity after 18 min) (Figure 5). [^68^Ga]Ga-BP-IDA uptake in the HCC was considerably lower than in non-cancerous tissue of the liver but higher than the background activity (Table 2, Figure 3).

Patient A demonstrated a newly diagnosed, well-differentiated HCC in the liver segments IVa/b, V, and VIII without vascular invasion. No extrahepatic metastases were detected. [^68^Ga]Ga-BP-IDA PET/CT was carried out for pre-interventional evaluation of the liver function distribution, given the large bilobar tumor and the possible damage to healthy liver tissue owing to the planned TARE treatment. It showed a high tumor-to-background ratio with high tracer uptake in the healthy, non-cirrhotic liver tissue (Figure 3). Tracer appeared in the bile ducts between 10 and 18 min after injection (Figure 4 and Figure 5). HCCs can retain a certain degree of metabolic and even excretory liver function, which can be relevant in patients with large tumors and with underlying diffuse liver damage (including, but not limited to, alcohol-related cirrhosis, non-alcoholic fatty liver disease, and hemochromatosis). However, in this case, based on the PET/CT, it was considered that the loss of remaining liver function in the tumor would not be prognostically relevant for the patient.

**Figure 6:**
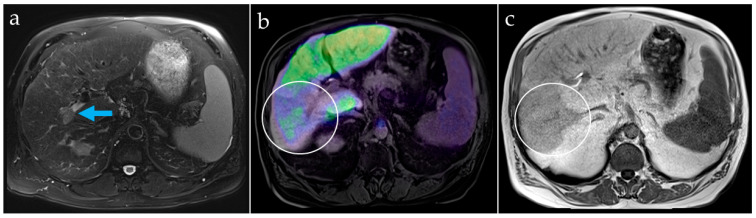
Patient B. Contrast-enhanced MRI of the liver in patient B shows tumor involvement and thrombus in the right portal vein branch ((**a**), arrow). The PET/MR fusion image (**b**) reveals liver areas with reduced [^68^Ga]Ga-BP-IDA uptake, corresponding to areas with liver texture disturbance on MRI (**c**), attributable to liver function impairment larger than the HCC lesions (white circles).

Patient B, also with newly diagnosed but partially necrotic HCC, showed homogenous hepatic uptake in the left liver lobe, but reduced uptake in liver segments V, VI, and VII, correlating with a changed signal in gadolinium-based contrasted MR imaging (Gd-EOB-DTPA, Primovist^®^, Bayer, Germany) that was attributed to a portal vein tumor thrombus and corresponding impairment of the tissue function (Figure 6). The tracer uptake in the non-tumorous, but cirrhotic liver tissue showed lower values than in patient A without liver cirrhosis, indicating impaired liver function (Table 1). Therefore, the tumor-to-liver ratio was lower. Gallbladder accumulation was seen early, after 10 min, suggesting sufficient biliary excretion.

## 4. Discussion

The radionuclide ^68^Gallium is an established positron emitter and commercially available as a generator product. It is able to undergo rapid complex formation with chelating agents containing hydroxy-, keto-, and carboxylic acid groups, allowing for the robust laboratory production of a wide range of radiopharmaceuticals [5]. Because of its physical half-life of 67.7 min, it is applicable in routine clinical diagnostics. BP-IDA, as an IDA-substituted tetrabromophthalein ligand, is also commercially available. Radiosynthesis of [^68^Ga]Ga-BP-IDA proved to be feasible and reproducible.

Bound to the transport protein albumin, IDA agents commonly used in hepatobiliary scintigraphy are carried to the liver and taken up by the hepatocytes by a carrier-mediated membrane transport mechanism (extraction phase) [6].They are excreted into the bile canaliculi (secretion phase) after intracellular passage and pass through the intra- and extrahepatic bile ducts into the small bowel (excretion phase), making them ideal tracers for biliary tract visualization [7,8]. Differences in the chemical structure between the different agents are the reason for differences in hepatic specificity and other features such as the ability to compete with bilirubin for hepatocyte uptake [9].

In a systematic comparison of the available IDA agents, the highest liver tissue uptake was seen for [^99m^Tc]Tc-mebrofenin, which also has a rapid hepatic uptake with a half-life in the blood pool of only 3–5 min [5,7]. Compared to the analysis performed by Schuhmacher et al., showing a [^68^Ga]Ga-BP-IDA liver uptake of up to 60% of the injected dose [4], hepatic uptake was lower in our study, with a maximum of 41.5%. This discrepancy might be the result of different quantification methods since a linear multicrystal positron scanner was used for absorption-corrected planar scintigraphy in 1983 instead of PET/CT volumetric quantification in our analysis. Besides, it is known that liver uptake of IDA agents can be affected by high plasma levels of bilirubin and hindered by hypoalbuminemia [10], neither of which was detected in our patient.

[^68^Ga]Ga-BP-IDA distribution in the liver, biliary system, and intestines in the first 60 min after tracer application is shown in Figure 2. In patient A, in whom whole-body imaging was performed, [^68^Ga]Ga-BP-IDA in the liver showed only a slow decrease in administered activity from 20 to 60 min after injection (from 41.5% to 37.4%), similar to the visual results of hepatic excretion in two healthy volunteers in 1983 [4]. At the time, the authors assigned this result to a longer tracer transit time in humans compared to experiments in rats, probably due to a higher release of ionic ^68^Ga after an enzymatic attack in the hepatocytes. Another reason could be the highly lipophilic character of the gallium complex, which decelerates hepatocellular transit time [11]. [^68^Ga]Ga-BP-IDA excretion from the liver may also be slowed down by high serum levels of bilirubin, since reported competitive action began at a serum level of 20 mg/dl, which was not present in our patient. The fastest liver transit has been shown for [^99m^Tc]Tc-mebrofenin, with an average hepatic excretion half time of 16 min [7]. A slow transit time might be unfavorable in biliary diagnostics since it reduces the bile-to-liver radioactivity ratio and thus degrades image quality [11].

In our patients, tracer began to accumulate in the gall bladder 18 min after tracer injection, which corresponds to standard values mentioned in the literature, with excretion phases starting after 15 to 30 min [12]. On account of its small organ size, activity in the gall bladder never exceeds 2.3% of the injected dose up to a time point of 60 min, which is similar to previous analyses [4], but shows increasing SUV_max_ up to 48.3% after 60 min due to tracer accumulation and concentration. Despite a slower hepatic transit time compared to [^99m^Tc]Tc-Mebrofenin [7], biliary excretion was visually clearly distinguishable within the first 30 min in two of three patients, making [^68^Ga]Ga-BP-IDA-PET/CT an interesting alternative to hepatobiliary scintigraphy in patients with suggested problematic biliary excretion. As expected, [^68^Ga]Ga-BP-IDA uptake in hepatocellular carcinoma was markedly lower than in non-tumorous liver tissue, presumably due to the alteration and destruction of hepatocytes.

Measurements showed no relevant urinary excretion of [^68^Ga]Ga-BP-IDA. As little as 0.5% of the injected activity was detected in the urinary bladder after 60 min, similar to [^99m^Tc]Tc-mebrofenin excretion (0.57%). For other IDA agents, higher urinary excretion rates ranging from 5–18% were demonstrated [1]. Due to the high lipophilic character of the gallium complex, urinary excretion of [^68^Ga]Ga-BP-IDA seems to be unaffected by high bilirubin serum levels, since the competitive action of bilirubin typically decreases biliary excretion and increases renal excretion of ^99m^Tc-IDA agents [4,13].

In general, to reach the optimal diagnostic potential, hepatobiliary agents should have high initial liver uptake and rapid biliary excretion [9]. Since [^99m^Tc]Tc-mebrofenin combines both features with a high hepatic uptake, fast excretion, and works better at higher bilirubin levels than other agents [7], in theory the application of [^68^Ga]Ga-mebrofenin seems most promising. However, efforts at complexing mebrofenin with Ga(III) have been undertaken, and it has been demonstrated that the non-modified IDA chelator mebrofenin is not a suitable ligand for ^68^Ga due to rapid demetallation [5,14].

Hepatobiliary imaging methods, in particular, are important for determining and quantifying liver function or future liver remnant, because global function parameters (measured for example via LiMAx test or indocyanine green clearance) cannot adequately depict the often heterogeneous distribution of liver function. While hepatobiliary magnetic resonance imaging (MRI) with liver specific contrast agents may be used for liver function quantification based on T1 relaxometry sequences, it has not yet found its way into clinical routine [15]. One reason for this is that in MRI, the signal caused by the contrast agent is always accompanied by an anatomical signal from the surrounding tissue, making quantification more complex. Therefore, a standard liver MRI protocol always requires two scans, one prior to contrast agent application and one following the administration of the agent [15]. It must be noted that the application of MRI contrast agents, which contain gadolinium chelates in high doses, does not come with insignificant risks [16,17]. In recent years, reports about gadolinium deposition and the occurrence of nephrogenic systemic fibrosis, particularly in MRI patients exhibiting impaired kidney function, have led to the marketing authorization of some MRI contrast agents being suspended by the European Medicines agency [18,19].

In contrast to MRI, nuclear imaging methods can use the radiotracer signal as a direct representation of liver function, making quantification much more feasible. However, while established hepatobiliary scintigraphy is a powerful technique providing visual information on both total and regional liver function, true quantification by scintigraphic methods is still limited [2]. Furthermore, in hepatobiliary scintigraphy, image acquisition is limited in terms of the combination of high temporal and high spatial resolution. Dynamic acquisition is commonly done in planar imaging mode since a three-dimensional scan takes several minutes, thus decisively decreasing temporal resolution. In contrast, in PET, both high temporal and high spatial acquisition can be combined. PET is a quantitative imaging modality by nature and is thus predestined to be used for quantitative hepatobiliary function imaging. Obviously, the necessity to inject a radioactive tracer and the resulting radiation exposure remain a drawback in nuclear medical imaging, compared to non-invasive imaging methods such as ultrasound. Nevertheless, the diagnostic information gained from the procedure usually justifies the risk.

Our image analyses showed that PET-based hepatobiliary function imaging allows identification of areas with impaired hepatocellular function, not only in tumorous tissue but also in demonstrating a decrease in tissue function downstream of a portal vein thrombosis. Compared with hepatobiliary scintigraphy, the higher spatial resolution of PET data can provide more detailed information about the localization of liver segments with impaired function. It can be used to estimate the remnant liver function after TARE since impairment of surrounding liver tissue is not completely avoidable, despite the possibility of a highly selective procedure. Additionally, pre- and postoperative information about biliary excretion and associated complications such as cholestasis, biliary obstructions, and bile leakage is given.

An important advantage of early dynamic PET with storing and processing list-mode data is the possibility of preserving the acquired spatial-temporal information that allows flexible, retrospective reconstruction of required time frames after the actual examination without increasing the radiation dose to the patient [20]. This provides the possibility of an additional reconstruction at 150 and 350 s after radiotracer injection for calculating the total hepatic uptake rate, similar to the methodology of hepatobiliary scintigraphy. This data should be assessed systematically in a follow-up study to show how regional hepatobiliary function can be quantified by PET/CT.

## 5. Conclusions

Hepatobiliary scintigraphy with ^99m^Tc-IDA agents is an established method in the evaluation of liver function and diagnostics of biliary diseases but has technical limitations regarding resolution and uptake quantification. Based on previous work, the GMP-compliant production of a ^68^Ga-labeled, IDA-substituted tetrabromophthalein ([^68^Ga]Ga-BP-IDA) was established and proved practicable and reliable. With the radiotracer, localization of areas with impaired liver function was possible. Additionally, the acquisition and storage of dynamic PET data allowing retrospective reconstruction of required time frames is a useful tool. This very early experience demonstrates that imaging with IDA-based PET tracers is feasible. It appears to be a promising tool for voxel-based assessment of hepatobiliary function with high temporal resolution. A prospective evaluation in a selected patient group should be performed to further assess the pharmacokinetic characteristics of the tracer and its clinical potential.

## Figures and Tables

**Table 1:** Quality control specifications of [^68^Ga]Ga-BP-IDA, given as mean values ± SD (*n* = 9).

Quality Control	Method	Acceptance Criteria	Result
Appearance	Visual inspection	Clear and colorless solution	Complies
pH	Potentiometric determination	5.0–8.0	6.5 ± 0.7
Ethanol content	Osmolarily measurement	≤10% (*v*/*v*)	6.1 ± 0.4%
HEPES content	TLC	≤200 µg/V, intensity of test solution spot similar or less than reference solution	Complies
Radionuclide identity	Half-life	62–74 min	68.0 ± 0.7 min
Radionuclide identity	Gamma-ray spectrometry	0.511 MeV and 1.077 MeV	Complies
Content of ^68^Ge (radionuclide purity)	Gamma-ray spectrometry	≤0.001%	2.8 × 10^−5^ ± 1.6 × 10^−5^%
Content of free ^68^Ga	Radio TLC	≤5.0%	2.6 ± 1.5%
Radiochemical purity	Radio HPLC	Activity assigned to [^68^Ga]Ga-BP-IDA ≥ 95.0%	97.4 ± 1.8%
Bacterial endotoxins	LAL test	≤10 IU/ml	0.5 ± 0.0 IU/mL
Sterility	Sterility testing	Sterile	Complies

**Table 2:** [^68^Ga]Ga-BP-IDA uptake in non-tumorous liver tissue and tumor tissue.

	Patient A (No Cirrhosis)	Patient B (Liver Cirrhosis)
Liver tissue (SUV_max_)	33.2	13.5
Tumor tissue (SUV_max_)	7.8	7.1
Tumor/liver ratio (SUV_max_)	4.3	1.9
Liver tissue (SUV_mean_)	23.5	8.3
Tumor tissue (SUV_mean_)	4.0	4.5
Tumor/liver ratio (SUV_mean_)	5.5	1.8

## Data Availability

Data sharing is not available for this article.
